# Adopting Text Mining on Rehabilitation Therapy Repositioning for Stroke

**DOI:** 10.3389/fninf.2019.00017

**Published:** 2019-03-19

**Authors:** Guilin Meng, Yong Huang, Qi Yu, Ying Ding, David Wild, Yanxin Zhao, Xueyuan Liu, Min Song

**Affiliations:** ^1^Shanghai Tenth People’s Hospital, School of Medicine, Tongji University, Shanghai, China; ^2^School of Informatics Computing and Engineering, Indiana University, Bloomington, IN, United States; ^3^School of Information Management, Wuhan University, Wuhan, China; ^4^School of Management, Shanxi Medical University, Shanxi, China; ^5^School of Informatics, Yonsei University, Seoul, South Korea

**Keywords:** text mining, ABC model, stroke, hand-arm bimanual intensive training, upper extremity

## Abstract

Stroke is a common disabling disease that severely affects the daily life of patients. Accumulating evidence indicates that rehabilitation therapy can improve movement function. However, no clear guidelines have specific and effective rehabilitation therapy schemes, and the development of new rehabilitation techniques has been relatively slow. This study used a text mining approach, the ABC model, to identify an existing rehabilitation candidate therapy method that is most likely to be repositioned for stroke. In the model, we built the internal links of stroke (A), assessment scales (B), and rehabilitation therapies (C) in PubMed and the links were related to upper limb function measurements for patients with stroke. In the first step, using E-utility, we retrieved both stroke-related assessment scales and rehabilitation therapy records and then compiled two datasets, which were called Stroke_Scales and Stroke_Therapies, respectively. In the next step, we crawled all rehabilitation therapies co-occurring with the Stroke_Therapies and then named them as All_Therapies. Therapies that were already included in Stroke_Therapies were deleted from All_Therapies; therefore, the remaining therapies were the potential rehabilitation therapies, which could be repositioned for stroke after subsequent filtration by a manual check. We identified the top-ranked repositioning rehabilitation therapy and subsequently examined its clinical validation. Hand-arm bimanual intensive training (HABIT) was ranked the first in our repositioning rehabilitation therapies and had the most interaction links with Stroke_Scales. HABIT significantly improved clinical scores on assessment scales [Fugl-Meyer Assessment (FMA) and action research arm test (ARAT)] in the clinical validation study for acute stroke patients with upper limb dysfunction. Therefore, based on the ABC model and clinical validation, HABIT is a promising repositioned rehabilitation therapy for stroke, and the ABC model is an effective text mining approach for rehabilitation therapy repositioning. The findings in this study would be helpful in clinical knowledge discovery.

## Introduction

Stroke is a common disabling health-care problem, and it is the second-leading cause of mortality and disability worldwide. In the United States, nearly 0.8 million people have stroke annually, and the estimated direct and indirect cost of stroke was $95 billion in 2015 and is expected to rise to 185 billion in 2030 (Brainin and Zorowitz, 2013; Cramer et al., 2017). The symptoms of acute stroke include physical impairments and cognitive dysfunction, and physical impairments of the affected limbs include movement restriction, sensory loss, and muscle activation abnormalities (Kuehn, 2018). About 50% of acute stroke survivors suffer from dysfunction of the upper limbs in the chronic phase (Favre et al., 2014), severely affecting the daily life and the therapeutic effect of rehabilitation therapy and reducing the quality of life of patients after stroke.

Rehabilitation therapies offer a chance for an individual to improve recovery and adapt to the new situation following acute stroke. Effective rehabilitation can help remodel the residual cortex, establish synaptic connections, and improve neurological function (Silva et al., 2018). A number of studies have investigated methods of rehabilitation management, including task-oriented training (Carrico et al., 2016), impaired limb forced training (Kwakkel et al., 2015), movement science-based therapy, robotic-assisted movement, virtual reality (VR) training (Brunner et al., 2017), functional electrical stimulation (Kattenstroth et al., 2018), and skill acquisition training paired with impairment mitigation and motivational enhancement. Small clinical studies have demonstrated that these rehabilitation methods can improve post-stroke dysfunction; however, no rehabilitation management is currently used as a part of the routine guideline practice, and the relative effectiveness of existing rehabilitation strategies has not been sufficiently evaluated in large clinical trials (Pollock et al., 2014). In addition, the quality, precision and focus, and risk of bias of these methods in these studies have not been assessed, and little breakthrough has been achieved with respect to intervention timing, dosage, biomarkers, standard assessment time points, and measures of current clinical practices. Furthermore, the stagnant development of new competitive rehabilitation strategies impedes rehabilitation development.

Therefore, the study of stroke rehabilitation has a long way to go. With the advent of the era of big data, new ideas can be provided for current standardized and personalized stroke rehabilitation strategies, and collaborative and multidisciplinary research programs need to be developed to provide new intervention choices for stroke rehabilitation with a standard universal rationale. Rehabilitation therapy repositioning provides new insight into possible unfolding data sources, such as a large amount of literature in PubMed, to mine appropriate new therapy strategies for stroke. The principal advantages of rehabilitation therapy repositioning over new therapy development are that the approved therapy has already been tested for safety, and repositioning can eliminate the time and cost of developing new therapies. Text mining can automatically extract information (facts or data) by systematically scrutinizing a vast number of abstracts or full-text versions of scientific publications (Percha and Altman, 2015; Meng et al., 2018a). Although there is no other published report on text mining in rehabilitation, some classic application examples can be found in the health science field (Westergaard et al., 2018). First, the publications in this field of text mining are growing at an exponential rate (Ding et al., 2013). Second, the medical literature is more standardized than other types of literature (He et al., 2011). Third, terms of the medical literature are relatively uniform and more convenient for mining. Typical text mining systems include the IBM Watson diagnosis system (Ahmed et al., 2017), DrugQuest (Papanikolaou et al., 2016), and disease-causing genes system (Sun et al., 2017).

This study aimed to examine the indirect relationship in the literature through the ABC model (Swanson and Smalheiser, 1997) to discover a promising rehabilitation strategy (rehabilitation repositioning). The ABC model has an internal connection among disease (A), assessment scale (B), and rehabilitation therapy (C). For stroke, most assessment scales of upper limb impairment assess a person’s ability to manage daily activities that require the use of the upper limbs; in addition, the rehabilitation therapies for functional improvement of upper limb share the same set of assessment scales, regardless of whether they are applicable for stroke or not. Therefore, we planned to identify undiscovered rehabilitation therapies for stroke through shared assessment scales and ultimately validate the most promising candidate in clinical settings.

## Materials and Methods

Our first focus was to find repositioning rehabilitation therapy candidates from an extensive collection of articles in PubMed. An early version of this study was published at Meng et al. (2018b). The present study describes our method in more detail. The codes and stroke-related PubMed data of these articles can be found in https://github.com/hyyc116/Stroke_findings/tree/master/DSTN. DSTN is the abbreviation of “disease scale test network.”

### ABC Model

**Figure 1:**
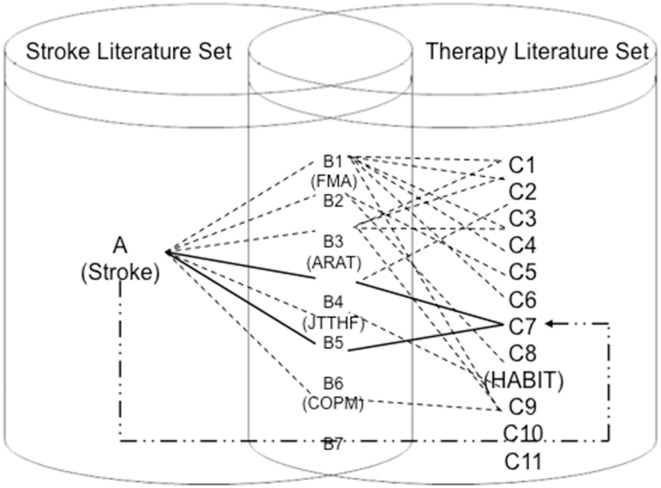
ABC model of Stroke—Assessment scales—Rehabilitation therapies …. Indicates a possible direct interaction between A (stroke), B (assessment scales), and C (therapies). __ Indicates a real direct interaction of stroke-assessment scales in this study. … Indicates the pathway of stroke and the targeted therapy hand-arm bimanual intensive training (HABIT) in this study.

In order to identify new rehabilitation therapies for stroke, we developed a relation extraction method based on ABC model, with which new knowledge could be discovered from sets of disjointed scientific entities, as shown in Figure 1. Figure 1 is a graphic representation of the ABC model adopted to discover rehabilitation therapy repositioning for Stroke. It illustrates that there are three different term sets: (1) stroke related terms extracted from Stroke Literature Set; (2) therapy related terms extracted from Therapy Literature Set; and (3) intermediate terms that occur in both literature sets. The connection among these three term sets is from Stroke to Therapy *via* intermediate terms (a.k.a. B terms). In particular, In Figure 1, dotted lines and solid lines represent a connection or a path from source to target terms. The solid line implies that it is a target discovery linked from Stork to Therapy *via* B terms whereas the dotted line is not of interest from the new discovery perspective.

The ABC model embodies the stages of retrieval, interpretation, and analysis. The retrieval phase investigates unstructured stroke-related information, designs text queries, and extracts the entity documents; the interpretation phase constructs a connected graph to represent A-B and B-C relationships between entities; in the analysis phase, annotation information between transitivity diffusion entities is used to sort the best entity that should be further verified. The ABC model starts with the theme “A” (stroke) in PubMed that collects scientific questions. The phrase “B” (assessment scale) is then listed, and a separate search is used for each “B” term; then, the phrase “C” (rehabilitation therapy) appearing in the code of the B is compiled; finally, with frequency criteria, the C terms are ranked such that a high ranked C term is used to represent the most promising hypothesis. For example, the term C may be the name of a therapeutic strategy that has not been tested for A (stroke) but has been demonstrated in other situations (e.g., in other forms of physical injury model or experimental animal model) with curative effects, suggesting that C may be explored as a new therapy.

### Study Procedure

**Figure 2:**
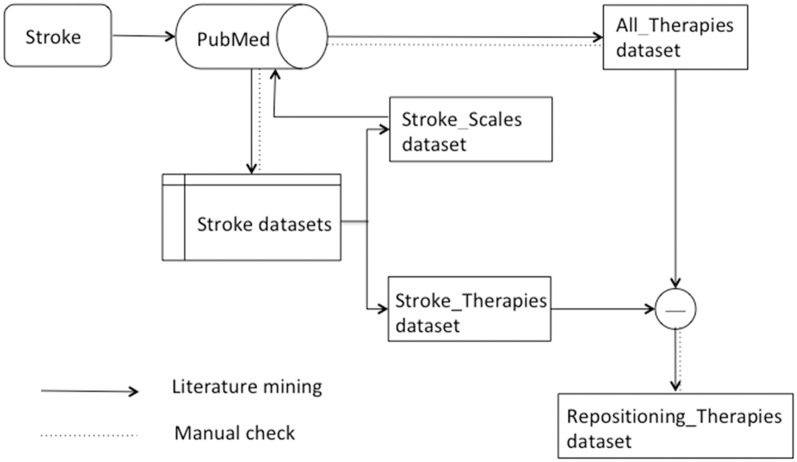
Flow chart from stroke to repositioning rehabilitation therapies. __Indicates text mining process in this study. … Indicates manual inspection in this study.

The overall flow chart is shown in Figure 2.

#### Stroke-Related Assessment Scales and the Creation of Rehabilitation Therapy Datasets

To collect articles related to stroke with upper limb impairment, we searched PubMed with stroke-related keywords (“stroke” OR “cerebral infarction” OR “brain ischemia” OR “cerebral hemorrhagic” OR “subarachnoid hemorrhage”) and (“hand” OR “arm” OR “upper extremity” OR “upper limb”) in April 2016. We did not use Medical Subject Headings (MeSHs) terms because most specific assessment scales and therapies do not directly belong to MeSH terms. When we searched stroke-related rehabilitation items, the MeSH terms that we obtained were only the common words, which should be deleted as confounding factors. The NCBI provides the E-utilities API for data query and download; therefore, we utilized python to call the API to fetch all query-related data in PubMed of NCBI through the HTTP protocol, and the data were then stored in plain text files (French et al., 2015). Scales and rehabilitation therapies are always used as noun phrases (NPs) in scientific articles. Thus, we applied a shallow chunk analyzer to extract NPs. The NPs ending with “test,” “scale,” “assessment,” “measure,” “score,” or “index” with frequency as the number of articles more than five were considered possible stroke assessment scale candidates. In addition, NPs ending with “training,” “therapy,” “treatment,” “treatments,” “practice,” “program,” “practise,” or “simulation” with frequency more than five were considered possible stroke therapy candidates. Besides frequency, the term frequency-inverse document frequency (TF-IDF) was also used as an index in this study. TF-IDF, a commonly used weighting technique for information retrieval and data mining, can be used to evaluate the importance of a phrase in a file; the importance of a phrase increases in proportion to the frequency it appears in the file. Because relying only on text mining could lead to false negatives, two senior neurologists (Liu and Zhao) examined the whole set independently and compared against each other to identify true positives as accurately as possible. The agreement rate of the two neurologists was as high as 96%, and the reconfirmation of the individual set with doubts reached a unified conclusion. In addition, they compared the results with the latest stroke rehabilitation guidelines of American Heart Association (Winstein et al., 2016) before stroke-related scale dataset (Stroke_Scales) and rehabilitation therapy dataset (Stroke_Therapies) were established.

#### Entire Rehabilitation Therapy Dataset Creation

With a direct stroke—assessment scales and therapies—assessment scales relationship, we crawled all therapies-related NP in PubMed co-occurring with Stroke_Scales *via* E-Utilities. The proceeding entire rehabilitation therapy dataset, named All_Therapies building, was similar to Stroke_Scales building mentioned above, and the NPs ending with “training,” “therapy,” “treatment,” “treatments,” “practice,” “program,” “practice,” or “simulation” with frequency more than five were kept to be possible therapy candidates, in which the assessment items were in Stroke_Scales. Manual inspection was the same as described above in Stroke_Therapies.

#### Potential Repositioning Rehabilitation Therapy Dataset

Therapies already included in Stroke_Therapies were deleted from All_Therapies so that the remaining therapies were not stroke-applied therapies, which could be repurposed for stroke. Manual inspection was then carried out again.

### Hypothesis Validation

#### Validation of the Retrieved Repositioning_Therapy Dataset in PubMed

The articles related to potential repositioning rehabilitation therapies and stroke-related keywords were retrieved from PubMed to ensure that no articles involve associations of therapies with stroke.

#### Further Rehabilitation Theory of Potential Candidate Exploration

The knowledge discovery is full of uncertainty and complicated. In the knowledge discovery, algorithms and methods could be perfect in theory, while the precision, recall, or some other metrics could be meaningless to some extended theories. This study aimed to find potential candidates. Therefore, clinical values of the potential candidates should be further comprehensively explored in the mechanism and principles of the rehabilitation theory.

#### Validation in Clinical Trial

A pre-clinical trial of adult acute stroke patients was carried out to test the rehabilitation effect by analyzing several sorts of data to clarify the potential advantage of rehabilitation therapy and determine the optimal rehabilitation approach for stroke patients.

## Results

### Text Mining Based on the ABC Model

We retrieved 11,418 records from PubMed with stroke-related keywords, and there were 10,992 records with abstracts. From this dataset, 241,044 unique NPs were extracted, including 81 different scales and 215 different rehabilitation therapies. In the potential scale list, the common phrases, such as “pre-test,” “post-test,” and “outcome assessment,” as well as other unrelated scales, such as “body mass index,” “depression score,” and “MMSE score,” were deleted after a manual check from the stroke-related scale dataset (Stroke_Scales), and 26 scales were finally obtained (Table 1). We ranked these assessment scales with frequency. The Fugl-Meyer assessment (FMA) had the highest frequency of 22.7%, followed by the Ashworth scale (AS), Barthel index (BI), action research arm test (ARAT), wolf motor function test (WMFT), Rankin scale (RS), functional independence measure (FIM), and primary outcome measure (POM) main outcome measure (MOM). Besides, the TF-IDF ranking was almost consistent with frequency. These scales are the most widely used assessment scales in stroke-related studies.

**Table 1:** Stroke_Scales dataset items with frequencies and term frequency-inverse document frequency (TF-IDF).

Abbreviations	Full name	Frequencies	TF-IDF
FMA	Fugl-meyer assessment	248	8.967532517
Ashworth	Ashworth scale	138	14.86100317
BI	Barthel index	115	21.46757397
ARAT	Action research arm test	81	12.07425711
WMFT	Wolf motor function test	75	13.60741523
RS	Rankin scale	55	19.33877467
FIM	Functional independence measure	42	15.96626141
MOM	Main outcome measure	41	5.37136245
MI	Motricity index	40	7.990027148
SIAS	Stroke impact scale	25	11.18520612
BBS	Berg balance scale	23	24.26706778
MAS	Motor assessment scale	22	14.7664421
RMA	Rivermead motor assessment	20	17.80426457
AAI	Arm index	14	12.81320984
BBT	Box and block test	14	9.018969872
FAT	Frenchay arm test	13	18.36081387
NIHSS	Nihss score	12	15.94629694
POM	Primary outcome measure	11	5.529408988
JTTHF	Jebsen-Taylor Test of Hand Function	8	15.01043442
DAS	Disability assessment scale	8	14.62921899
BRUNSTROM	Brunnstrom scale	7	9.218846945
COPM	Canadian occupational performance measure	7	13.53841027
NHPT	Nine hole peg test	7	16.42409311
VAS	Visual analog scale	7	7.112188973
RS	Rankin scale	7	19.33877467
AMAT	Arm motor ability test	6	10.48555474

Accordingly, in the potential therapy list, the following common words were deleted: “clinical practice,” “conventional therapy,” “medical therapy,” “physical therapy,” “specific training,” and “combined therapy”; in addition, the following drug therapies were deleted in the manual inspection process: “antiplatelet therapy,” “anticoagulant therapy,” “antihypertensive therapy,” and “antithrombotic therapy.” Subsequently, we obtained the stroke-related rehabilitation therapy dataset, Stroke_Therapies, which compromised 47 rehabilitation therapies (Table 2). Two senior neurologists conducted the manual inspection independently, and we also compared our results with those in one recent review, which focused on stroke rehabilitation assessment (Santisteban et al., 2016). It was found that our data of Stroke_Scales and Stroke_Therapies were consistent with those in the previous review.

**Table 2:** Stroke_Therapies dataset items with frequencies.

Stroke_Therapy items	Frequencies	Stroke_Therapy items	Frequencies
Transcranial magnetic stimulation	495	Treadmill training	15
Electrical stimulation	296	Peripheral nerve stimulation	14
Induced movement therapy	251	Bimanual training	13
Robot therapy	125	Gait training	13
Mental practice	97	Motor imagery training	13
Mirror therapy	97	Computer interface training	12
Intensive occupational therapy	95	Smart arm training	12
Motor training	74	Massed practice	10
Somatosensory stimulation	60	Transcutaneous electrical nerve stimulation	10
Repetitive practice	48	Virtual reality training	10
Intensive training	44	Physical and occupational therapy	9
Neuromuscular electrical stimulation	40	Active neuromuscular stimulation	8
Bilateral training	32	Deep brain stimulation	7
Bilateral arm training	29	Functional strength training	7
Noninvasive brain stimulation	27	Functional task practice	7
Cortical stimulation	24	Motor cortex stimulation	7
Tactile stimulation	24	Music therapy	7
Upper extremity training	24	Wrist training	7
Hand training	23	Based mental practice training	6
Neuromuscular stimulation	22	Constraint-induced movement therapy	6
Median nerve stimulation	20	Forced use therapy	6
Task practice therapy	19	Paired associative stimulation	6
Aerobic exercise training	17	Surface neuromuscular electrical stimulation	6
Unilateral training	17		

**Figure 3:**
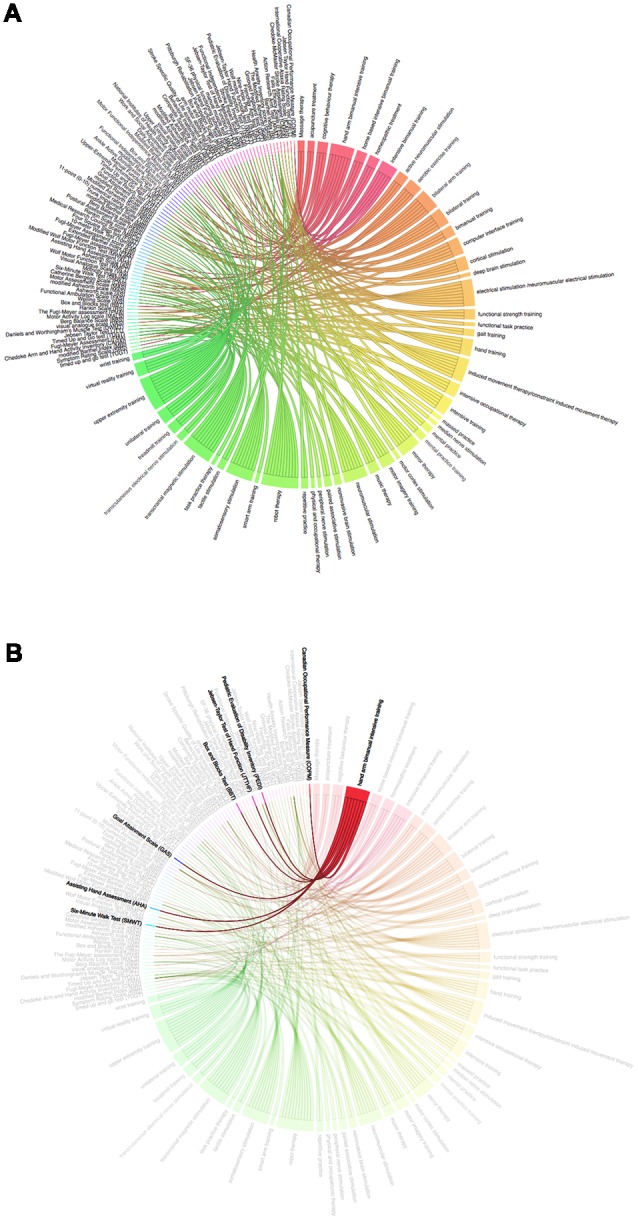
Interactions of assessment scales and therapies. **(A)** Interactions of assessment scales and all therapies. **(B)** Interaction of HABIT and assessment scales.

Using the extracted Stroke_Scales, we searched again in PubMed and retrieved 60,307 records, in which 60,202 records had abstracts. From these records, we extracted the rehabilitation therapies (All_Therapies dataset), removed those applied for stroke and listed in Table 2 (Stroke_Therapies dataset), and obtained the potential repositioning rehabilitation therapies for stroke (Table 3). The interactions between Stroke_Scales and All_Therapies datasets are shown in Figure 3.

**Table 3:** Potential repositioning rehabilitation therapies.

Unknown rehabilitation therapies	Frequencies	Unknown rehabilitation therapies	Frequencies
Cognitive behavior therapy	146	Acupuncture treatments	12
Massage therapy	30	Hand arm bimanual intensive training	7
Homeopathic treatment	16		

As shown in the upper left quadrant in Figure 3, Stroke_Scales were densely distributed and were interacted with the All_Therapies dataset from the other three quadrants. Among All_Therapies, the potential repositioning rehabilitation therapies were marked in red and rose, while the existing Stroke_Therapies dataset items were indicated in other colors. Among the potential repositioning rehabilitation therapies, “hand arm bimanual intensive training” had the most interactions with Stroke_Scales, including Jebsen-Taylor Test of Hand Function (JTTHF), canadian occupational, performance measure (COPM), Assisting Hand Assessment (AHA), Pediatric Evaluation of Disability Inventory (PEDI), Box and Blocks Test (BBT), Six-Minute Walk Test (SMWT), and Goal Attainment Scale (GAS), none of which was the commonly used stroke-related assessment scale according to targeted populations and discipline difference between adult stroke and pediatric cerebral palsy.

We used these potential repositioning rehabilitation therapies with “stroke” in PubMed search to exclude the records that include the associations of those rehabilitation therapies with stroke. We found that except for “hand arm bimanual intensive training,” other NPs all co-occur with stroke. Thus, the “hand arm bimanual intensive training” was our target validation candidate.

### Hand-Arm Bimanual Intensive Training (HABIT)

Hand arm bimanual intensive training (HABIT) is a bimanual intervention addressing the specific upper extremity impairments in pediatric congenital hemiplegia, which is the most common physical disability in childhood (Charles and Gordon, 2006; Gordon et al., 2007), typically with impairments of spasticity, sensation, and reduced strength. HABIT has been found to improve the pediatric patients’ bimanual hand coordination and the space control of actions. Furthermore, HABIT has been demonstrated to be the prioritized optimal approach to improve bimanual hand use and activity performance for children with hemiplegia (Green et al., 2013), and its principles include motor learning (practice specificity, types of practice, and feedback) and improved neuroplasticity (practice-induced brain changes arising from repetition, increased movement complexity, motivation, and reward), which are also the critical contents of stroke rehabilitation functional goals.

We checked the frequency of different scales for stroke and found that compared with JTTHF and COPM, FMA was the more commonly used measure for the upper limb in the adult population, accounting for almost 30% of the total frequencies (Table 2). The findings are consistent with those of a systematic review of stroke rehabilitation (Santisteban et al., 2016). Based on the fact that FMA is usually applied in combination with ARAT in clinical studies, we decided to apply FMA combined with ARAT, not COPM or JTTHF, in our clinical validation study.

The validation of the potential candidate of repositioning rehabilitation therapies was examined in our little preclinical trial of stroke patients with upper limb impairment. The trial was approved by the local ethics committee of Shanghai Tenth People’s Hospital. Patients adequately understood the purpose of the study, agreed to participate in this study, and signed the informed files. A total of 10 patients with acute stroke were recruited and randomly divided into two groups: the routine rehabilitation program (RRP) and the HABIT groups. The same therapist who held national certification performed all treatments and training. The treatment duration was 2 weeks (10 working days), and the rehabilitation time was the same in both groups. In general, motor function and extremity activity were assessed using FMA and ARAT before and after therapy in these patients. Both groups showed statistically significant improvements of FMA and ARAT from baseline to post-treatment assessment. While more participates need to be enrolled to get a positive comparison between HABIT group and RRP group (Table 4). More details were described in our follow-up full clinical trial study published in Frontiers in Neurology (Meng et al., 2018c).

**Table 4:** Clinical validation of HABIT on patients with acute stroke.

HABIT group (*n* = 5)					RRP group (*n* = 5)				
	FMA		ARAT			FMA		ARAT	
No.	Baseline	Post-training	Baseline	Post-training	No.	Baseline	Post-training	Baseline	Post-training
#1	32	36	32	34	#1	30	33	31	33
#2	35	45	33	38	#2	32	33	31	34
#3	33	36	31	33	#3	32	34	29	30
#4	33	37	30	33	#4	37	39	33	34
#5	29	36	34	37	#5	33	34	32	34

*RRR, the routine rehabilitation program; HABIT, Hand-arm bimanual intensive training; FMA, Fugl-Meyer assessment; ARAT, action research arm test; *p*(FMA, baseline, HABIT vs. RRP) = 0.798815616; *p*(FMA, post-training, HABIT vs. RRP) = 0.148803893; *p*(ARAT, baseline, HABIT vs. RRP) = 0.433301901; *p*(ARAT, post-training, HABIT vs. RRP) = 0.16682164; *p*(HABIT group, FMA, baseline vs. post-training) = 0.030637606; *p*(HABIT group, ARAT, baseline vs. post-training) = 0.049413148*.

## Discussion

Stroke is caused by the sudden rupture of blood vessels in the brain or blood vessels that cannot flow into the brain owing to vascular occlusion. Pediatric congenital hemiplegia mostly occurs because of poor nutrition during pregnancy, anemia during pregnancy, encephalopathy, birth asphyxia, pathological jaundice, and intrauterine distress, causing immature fetal brain and other organ tissues. Furthermore, pediatric congenital hemiplegia can further damage the central nervous system, leading to cerebral palsy. Although the pathogenesis and clinical manifestations of stroke and pediatric congenital hemiplegia are not the same, the HABIT is effective for both conditions, indicating that the two diseases share similar rehabilitation mechanisms. Besides, patients who received HABIT showed significant improvement, indicating that HABIT has a positive impact on rehabilitation therapy for upper extremity impaired patients. The principle of HABIT includes motor learning (task specificity, task type, and feedback), neuroplasticity, brain transformation upon increasingly difficult therapy and incentive reward (Nudo, 2003), and training cortical-spinal system reconstruction [manifesting as function recovery after injury (Eyre, 2003)] and is the theoretical foundation underlying the hypothesis that bimanual intensive therapy could improve the upper extremity function after acute stroke.

Although the repositioning of rehabilitation therapy is common in clinical practice, the discovery is often based on the clinician’s personal experience or deduction from the medical community, without objective and systemic approaches with data mining application. For instance, VR, a rehabilitation intervention, was first applied to basic motor disability (Greenleaf and Tovar, 1994). Studies in the area of VR-based rehabilitation have gained growing recognition of the potential value of VR for other diseases with motor disorders, such as Parkinson’s disease. VR is presently proposed as a new rehabilitation tool that potentially optimizes motor learning in a safe environment and replicates real situations to help improve functional activities in daily life such as gait, balance, and quality of life (Dockx et al., 2016). In our study, stroke (A) was associated with the assessment scales (B) in stroke literature, and in the rehabilitation therapy literature, assessment scales (B) represented the effect of rehabilitation therapy (C). It is highly likely that the retrieved rehabilitation therapies, which are unknown for stroke, have positive effects on stroke. In recent years, bioinformatics mining and omics studies have indirectly utilized the model (Smalheiser, 2017). To the best of our knowledge, this study is the first to use the ABC model to show the repositioning of rehabilitation therapy with positive validation. This model could be generalized as disease-assessment scale-rehabilitation therapy in future studies.

However, there is a major concern with the approach taken in the study. The HABIT prototype had started earlier than text mining was adopted. In the beginning, the clinicians generated a hypothesis that HABIT could be re-positioned in stroke rehabilitation and prepared to apply for the clinical ethics application. In order to avoid uncertainty in the trial, the clinicians sought cooperation from information scientists, hoping to find more supporting evidence from existing PubMed literatures using advanced text mining algorithms, and this is why the clinical research was not immediately carried out after the application of clinical ethics. Later, researchers in the two domains (clinical research and text mining) interacted with each other on numerous occasions, constantly trying to adjust the parameters, and finally provided effective evidence for HABIT. Besides, we relied much on a manual check, more than half were deleted after the manual check.

We understand that knowledge discovery is a process of trial and error, and it is an iterative procedure that domain scientists work closely with information scientists. The present study has shown the potential of applying text mining for new medical discoveries. Mining literatures to generate a hypothesis about rehabilitation therapy repositioning semi-automatically could be the *de facto* approach to inform clinicians who are trying to master the exponentially rapid expansion of publications and datasets. As demonstrated in this study, the ABC model can be applied to new hypothesis generation for rehabilitation therapy repositioning.

## Conclusion

In the present study, we proposed a text mining approach to mine terms related to disease, rehabilitation therapy, and assessment scale from literature, and a subsequent ABC inference analysis was used to identify relationships of these terms across publications. The clinical validation study demonstrated that our approach could be used to identify potential repositioning rehabilitation therapy strategies for stroke. In particular, we identified a promising rehabilitation method, called HABIT, which was previously used in pediatric congenital hemiplegia. A subsequent full clinical trial confirmed the HABIT as a highly promising rehabilitation therapy for stroke. Further clinical studies are needed to investigate the long-term effect of HABIT on stroke patients and identify optimal parameters of the therapy. In addition, further studies are needed to improve text mining and inference strategy and identify other clinical applications amenable to the technique.

## Author Contributions

GM, XL, and MS designed the study. YH and YD performed the experiments and prepared the figures. QY, DW, and YZ analyzed the data. XL and YZ performed the manual inspection as senior domain experts. GM wrote and discussed all sections of the manuscript. All authors reviewed and approved the manuscript.

## Conflict of Interest Statement

The authors declare that the research was conducted in the absence of any commercial or financial relationships that could be construed as a potential conflict of interest.
